# Evodiamine Synergizes with Doxorubicin in the Treatment of Chemoresistant Human Breast Cancer without Inhibiting P-Glycoprotein

**DOI:** 10.1371/journal.pone.0097512

**Published:** 2014-05-15

**Authors:** Shengpeng Wang, Lu Wang, Zhi Shi, Zhangfeng Zhong, Meiwan Chen, Yitao Wang

**Affiliations:** 1 State Key Laboratory of Quality Research in Chinese Medicine, Institute of Chinese Medical Sciences, University of Macau, Macau, China; 2 Department of Cell Biology & Institute of Biomedicine, College of Life Science and Technology, Jinan University, Guangzhou, Guangdong, China; University of South Alabama, United States of America

## Abstract

Drug resistance is one of the main hurdles for the successful treatment of breast cancer. The synchronous targeting of apoptosis resistance and survival signal transduction pathways may be a promising approach to overcome drug resistance. In this study, we determined that evodiamine (EVO), a major constituent of the Chinese herbal medicine *Evodiae Fructus*, could induce apoptosis of doxorubicin (DOX)-sensitive MCF-7 and DOX-resistant MCF-7/ADR cells in a caspase-dependent manner, as confirmed by significant increases of cleaved poly(ADP-ribose) polymerase (PARP), caspase-7/9, and caspase activities. Notably, the reversed phenomenon of apoptosis resistance by EVO might be attributed to its ability to inhibit the Ras/MEK/ERK pathway and the expression of inhibitors of apoptosis (IAPs). Furthermore, our results indicated that EVO enhanced the apoptotic action of DOX by inhibiting the Ras/MEK/ERK cascade and the expression of IAPs without inhibiting the expression and activity of P-glycoprotein (P-gp). Taken together, our data indicate that EVO, a natural product, may be useful applied alone or in combination with DOX for the treatment of resistant breast cancer.

## Introduction

One of the most serious problems responsible for the failure of cancer chemotherapy in the clinic is the occurrence of multidrug resistance (MDR). Cancer cells can exhibit resistance to multiple functionally different agents, such as vinca alkaloids, anthracyclines and taxanes, after treatment with one type or class of drugs [1]. In recent decades, the mechanisms responsible for MDR have been heavily investigated, although the available treatment options in the clinic remain limited [2]. It has been suggested that increased drug efflux by one or more energy-dependent transporters, including P-glycoprotein (P-gp), multidrug resistance associated proteins (MRPs), and breast cancer resistance protein (BCRP), and changes in the targeted enzymes, altered cell cycle checkpoints and anti-apoptotic mechanisms could all result in MDR [3].

Apoptosis resistance and increased survival signaling are the major regulators of cancer cell survival against chemotherapy, and targeting only one of these pathways may be insufficient to obtain chemotherapeutic effects [4]. The inhibitors of apoptosis (IAPs), an effective group of conserved endogenous proteins that includes X-linked inhibitor of apoptosis (XIAP), survivin and cellular inhibitor of apoptosis protein 1 and 2 (cIAP1 and cIAP2), can inhibit apoptosis by binding to caspase-9 and the downstream caspase-3 and caspase-7 in the intrinsic apoptosis pathway [5]–[7]. The overexpression of IAPs has been demonstrated to confer protection against apoptosis in several resistant cancers [8], [9]. Targeting IAPs to release caspases and subsequently activate apoptosis has become a popular strategy in designing novel drugs to conquer MDR. Meanwhile, IAP antagonists have been shown to enhance the chemotherapeutic effect of different classes of cytotoxic drugs against various cancers [10].

Survival signals help cells to overcome stressful or deleterious stimuli by increasing the expression or activity of many survival factors [4]. The Ras/MEK/ERK cascade is one of the key signaling pathways involved in the regulation of cell cycle progression, growth and differentiation [11], [12]. Activation of the Ras/MEK/ERK signaling can alter the expression, activity and subcellular localization of many proteins that play key roles in apoptosis, and has been associated with resistance to chemotherapeutic agents in many cancers [13]. Chemotherapeutic agents can activate the Ras/MEK/ERK pathway by diverse mechanisms, including ROS-induced calmodulin-dependent kinase (CaM-K) activation [14]. Chemotherapeutic drugs such as doxorubicin (DOX) can also activate p53 to increase the expression of the discoidin domain receptor (DDR), which in turn activates the Ras/MEK/ERK pathway [13]. Therefore, synchronously targeting IAPs and survival signal transduction pathways may be a promising approach to overcome drug resistance.

Evodiamine (EVO), a naturally occurring indole alkaloid, is one of the main bioactive components of the herbal medicine *Evodia rutaecarpa* (Juss.) Benth. [15]. EVO has been shown to exhibit anti-tumor properties by suppressing tumor growth [16], metastasis [17] and angiogenesis [18]. Furthermore, EVO can induce apoptosis by inhibiting nuclear factor kappa B (NF-κB) activation, leading to the downregulation of NF-κB-regulated gene products such as Cyclin D1, XIAP, Bcl-2, and Bcl-XL [19], [20]. The PI3K/Akt and ERK signaling pathways also play important roles in cancer cell apoptosis in responses to EVO [21]-[23]. The objective of the present study was to determine the effects of EVO on DOX-resistant breast cancer cells when treated alone and in combination with DOX. We hypothesized that EVO would enhance DOX sensitivity in DOX-resistant breast cancer cells by synchronously inhibiting IAPs and survival signal transduction pathways. Our results indicated that EVO induced apoptosis of both DOX-sensitive and DOX-resistant cells and enhanced the apoptotic action of DOX by inhibiting both IAPs and the Ras/MEK/ERK cascade without inhibiting P-glycoprotein (P-gp).

## Materials and Methods

### Reagents

EVO (98% purity) was purchased by Sigma-Aldrich. DOX (98% purity) was obtained from Meilun Biology Technology Company (Dalian, China). Dulbecco's Modified Eagle Medium (DMEM), fetal bovine serum (FBS), penicillin-streptomycin (PS), phosphate-buffered saline (PBS), propidium iodide (PI) and 0.25% w/v trypsin/1 mM EDTA were purchased from Gibco Life Technologies (Grand Island, USA). The lactate dehydrogenase (LDH) release detection kit was obtained from Roche Diagnostics. Hoechst 33342 and 3-[4,5-dimethyl-2-thiazolyl]-2,5-diphenyl tetrazolium bromide (MTT) were obtained by Molecular Probes (Grand Island, USA). Primary antibodies against cleaved caspase-7, cleaved caspase-9, cleaved PARP, Ras, phosphorylated MEK, MEK, phosphorylated ERK1/2, ERK1/2, XIAP, cIAP1, survivin, P-gp and GAPDH and secondary antibodies were purchased from Cell Signaling Technology.

### Cell Lines and Cell Culture

MCF-7 human breast cancer cells were obtained from the American Type Culture Collection (ATCC). The DOX-resistant MCF-7/ADR cells were obtained from stepwise exposure to increasing concentrations of DOX as originally described [24]. Cells were cultured in DMEM medium with antibiotics (100 µg/ml streptomycin, 100 U/ml penicillin) and heat-inactivated 10% (v/v) FBS at 37°C in a humidified atmosphere of 5% CO_2_.

### MTT Assay and LDH Assay

The colorimetric MTT assay was modified and executed to quantify cell proliferation [25]. Exponentially growing MCF-7 and MCF-7/ADR cells were seeded in 96-well plates at a final concentration of 5×10^3^ cells/well. After incubation for 24 h, cells in designated wells were treated with different concentrations of EVO. After 24, 48 and 72 h incubation, cell viability was detected by with the addition of free serum DMEM medium containing 1 mg/ml MTT for 4 h and subsequently dissolving the formed formazan crystals with DMSO. The absorbance in each individual well was determined at 570 nm by microplate reader (SpectaMax M5, Molecular Devices). The proliferation rates of cancer cells were evaluated by using triplicate assays. The LDH release rates from cells were evaluated by a commercial kit according to the manufacturers' protocol (Roche).

### Analysis of Nuclear Morphology

MCF-7 cells and MCF-7/ADR cells were treated with different doses of EVO for 24 h. After treatment, cells were washed twice with PBS and fixed with 4% paraformaldehyde for 20 min. After incubation with Hoechst 33342 (5 µg/ml) at room temperature for 15 min, cells were observed by Incell Analyzer 2000 (GE Healthcare Life Sciences, USA) to survey the apoptotic morphology of the cell nucleus of MCF-7 cells and MCF-7/ADR cells. Condensed, fragmented or degraded nuclei indicated apoptosis in MCF-7 and MCF-7/ADR cells, and the results were based on at least three independent experiments.

### Annexin V/PI Staining Assay

Apoptotic cells were detected by an Annexin V-FITC/PI apoptosis detection kit (BioVision) according to manufacturer's instruction. MCF-7 cells and MCF-7/ADR cells were treated with different concentrations of EVO. After 48 h of incubation, cells were trypsinized and collected by centrifugation at 500 g/min for 5 min. After being washed twice with cold PBS and gently suspended in 100 µl binding buffer, cells were stained with 5 µl of Annexin-FITC and 10 µl of PI solution and incubated in the dark at room temperature for 15 min. Cell apoptosis was analyzed by a flow cytometer (BD Biosciences). All experiments were performed in triplicate.

### Caspase Activity Assay

Caspase-Glo assay kits (Promega) were used to measure the caspase activities according to the manufacturer's instructions. MCF-7 cells and MCF-7/ADR cells were plated into 96-well white-walled plates (PerkinElmer). Twenty-four hours after seeding, cells were treated with different concentrations of EVO for 48 h. Subsequently, 100 µl of caspase-3/7 or caspase-9 assay reagent was added to each well, and the plate was incubated in the dark for 1 h. The luminescence was measured by using a SpectraMax M5 microplate reader (Molecular Devices). Caspase activity was expressed as a percentage of the untreated control treatment (DMSO). All samples were assayed in triplicate.

### Western Blotting

MCF-7 cells and MCF-7/ADR cells were treated with different concentrations of EVO for 48 h, and the total protein was extracted with RIPA lysis buffer containing 1% phenylmethanesulfonylfluoride (PMSF) and 1% protease inhibitor cocktail. As previously reported, protein concentrations were determined with a BCA protein assay kit (Thermo Scientific) [26]. Equivalent amounts of proteins from each group were separated by SDS-PAGE and were transferred to PVDF membranes. After being blocked for 1 h with 5% non-fat dried milk, the membranes were incubated with specific primary antibodies (1: 1000) and subsequently incubated with the corresponding secondary antibodies (1: 1000). An ECL Advanced Western Blotting Detection kit (GE Healthcare) was used to visualize and detect the specific protein bands. All band densitometries were calculated using Quantity One Software (Bio-Rad).

### Combination Studies of EVO and DOX

Exponentially growing MCF-7 cells and MCF-7/ADR cells (5×10^3^) were seeded in 96-well plates and incubated for 24 h. Cells were pretreated with different concentrations of EVO for 12 h and incubated with the indicated concentrations of DOX for another 48 h. Cell viability was measured by MTT assay as described previously. Apoptotic cells were detected by Annexin V-FITC/PI dual staining as described previously.

### Determination of Intracellular DOX

MCF-7 cells and MCF-7/ADR cells were pretreated with different concentrations of EVO. After 12 h incubation, the media were removed, and the cells were washed two times with PBS. Cells were then incubated with different concentrations of DOX for an additional 4 h. Extracellular DOX was removed, and the cells were collected and washed with PBS three times. The fluorescence of intracellular DOX was analyzed by flow cytometry (BD Biosciences). The corresponding single parameter histogram of the fluorescence signal of the collected cells (1.0×10^4^) was amplified and generated by using FlowJo software.

### P-gp Activity Assay

A fluorometric MDR assay kit (Abcam, Cambridge, UK) was used to determine the activity of P-gp. Following the user protocol provided in the fluorometric MDR assay kit, MCF-7 cells and MCF-7/MDR cells (1.0×10^4^ cells/well) were seeded into 96-well flat clear-bottom black-wall microplates and incubated for 24 h. The cells were treated with different concentrations of EVO for 12 h. Next, 100 µl MDR dye-loading solution was added to each well and incubated at 37°C for 1 h avoiding light. Intracellular fluorescence was detected using a microplate reader (SpectraMax M5, Molecular Devices, USA) at an excitation wavelength of 490 nm and an emission wavelength of 525 nm. All experiments were performed in triplicate and compared to controls.

### Statistical Analysis

All data were presented as means ± SE. Each value exhibited the mean of at least three independent experiments in each group. In all cases, Student's t-test was used for statistical comparison. P values less than 0.05 were considered significant.

## Results

### The Effect of EVO on Proliferation and Cytotoxicity in DOX-Sensitive and DOX-Resistant Breast Cancer Cells

**Figure 1:**
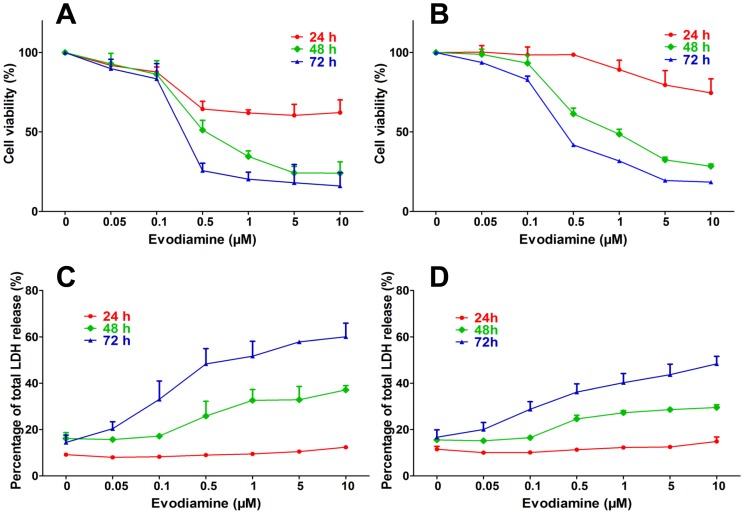
Effects of different concentrations of EVO on the proliferation of Dox-sensitive MCF-7 (A) and Dox-resistant MCF-7/ADR (B) cells by MTT assay. The cytotoxicity caused by different concentrations of EVO in MCF-7 (**C**) and MCF-7/ADR (**D**) cells was determined by LDH assay. Each point represents the mean ± SE.

We first assessed the effect of EVO on the proliferation of MCF-7 and MCF-7/ADR cells. As shown in **Figure 1A and 1B**, EVO exhibited time- and concentration-dependent inhibitory effects on both MCF-7 and MCF-7/ADR cells. The IC_50_ values of MCF-7 cells treated with EVO for 24, 48, and 72 h were 7.68 µM, 0.64 µM and 0.30 µM, respectively. The IC_50_ values of the MCF-7/ADR cells were higher than those for MCF-7, with values of 24.47 µM, 1.26 µM and 0.47 µM for 24, 48 and 72 h treatment of EVO, respectively. The cytotoxicity induced by EVO in MCF-7 and MCF-7/ADR cells was determined by LDH assay. The LDH released profiles of MCF-7 (**Figure 1C**) and MCF-7/ADR (**Figure 1D**) cells exposed to EVO for 24 h were nearly equivalent, whereas higher levels of LDH were released by MCF-7 cells after treatment with EVO for 48 h and 72 h compared with MCF-7/ADR cells, which was consistent with the results of the MTT assay.

### EVO Induced Apoptosis in MCF-7 and MCF-7/ADR Cells

**Figure 2:**
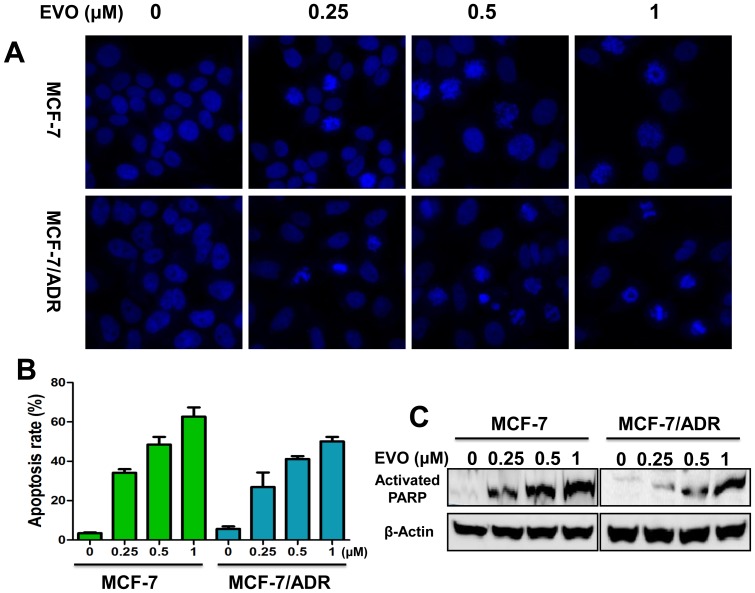
EVO induces apoptosis in MCF-7 and MCF-7/ADR cancer cells. (**A**) Hoechst 33342 fluorescent staining to detect apoptotic morphology of MCF-7 and MCF-7/ADR cells after treatment of different concentrations of EVO for 48 h. Apoptotic cells were recognized by condensed, fragmented and or degraded nuclei. Cells were observed of three experiments using Incell Analyzer 2000 (GE healthcare) (**B**) Quantitative apoptotic measurement by Annexin V/PI double staining in MCF-7 and MCF-7/ADR cells after treatment of different concentrations of EVO for 48 h. Data were expressed as mean ± SE of three independent experiments. * P<0.05 vs. untreated control (MCF-7), ^#^ P<0.05 vs. untreated control (MCF-7/ADR). (**C**) MCF-7 and MCF-7/ADR cells were plated on 100 mm-diameter dishes and treated with different concentrations of EVO for 48 h. The cells were used for Western blot analysis using antibodies against activated PARP and β-Actin.

Hoechst staining revealed marked nuclear morphological changes in MCF-7 and MCF-7/ADR cells after EVO treatment, including the condensation of chromatin and nuclear fragmentation (**Figure 2A**). To further confirm the results, we performed Annexin V/PI double staining to quantitatively detect apoptosis. As shown in **Figure 2B**, after 48 h of treatment, EVO (1 µM) induced apoptosis in 62.7% and 50.1% of MCF-7 and MCF-7/ADR cells, respectively. PARP is a protein involved in many cellular processes as DNA repair and cell apoptosis [27], and cleaved PARP is a marker of apoptosis. EVO treatment increased the expression of cleaved PARP in the present study in a concentration-dependent manner (**Figure 2C**). Taken together, these results revealed that the inhibitory effects of EVO on the proliferation of MCF-7 and MCF-7/ADR cells were caused by the induction of apoptosis.

### The Effects of EVO on Caspase Processing

**Figure 3:**
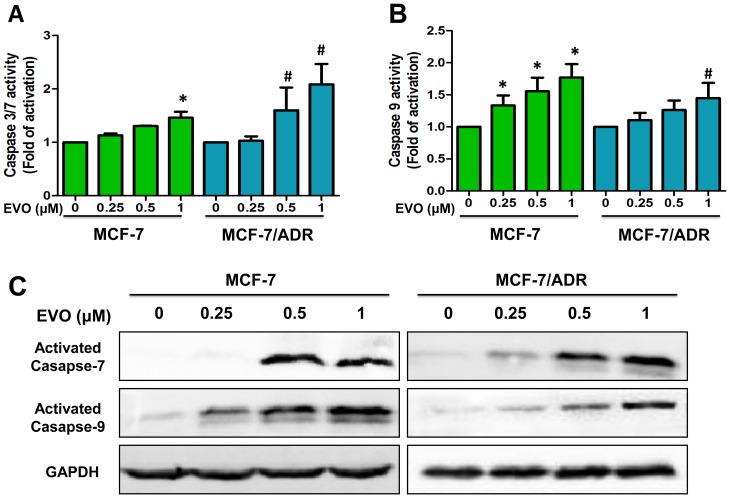
Involvement of caspase activation in MCF-7 and MCF-7/ADR cells after treated with EVO for 48 h. EVO increase the activities of caspase 3/7 (**A**) and caspase 9 (**B**) in MCF-7 and MCF-7/ADR cells by a dose-dependent manner. Data were expressed as mean ± SE of two or three independent experiments. * P<0.05 vs. untreated MCF-7 cells, ^#^ P<0.05 vs. untreated MCF-7/ADR cells. (**C**) MCF-7 and MCF-7/ADR cells were treated with different concentrations of EVO for 48 h. The cells were used for Western blot analysis using antibodies against activated Caspase 7, 9 and GAPDH. Similar results were obtained in two or three separate experiments.

As EVO induced MCF-7 and MCF-7/ADR cell apoptosis, the mechanism of apoptosis induced by EVO was further investigated. Caspases exist as inactive zymogens in normal cells and undergo proteolytic processing upon activation during apoptosis [28]. As caspases are responsible for the execution of apoptosis, we further examined caspase activation using caspase activity kits (Promega). The data confirmed that the caspase-7 activities (**Figure 3A**) of MCF-7 and MCF-7/ADR cells were increased by 1.46-fold and 2.08-fold after 48 h EVO (1 µM) treatment compared with control cells, and the caspase-9 activities (**Figure 3B**) were increased by 1.77-fold and 1.45-fold in MCF-7 and MCF-7/ADR cells, respectively. We then explored whether caspases were processed and cleaved during the course of EVO-induced apoptosis. MCF-7 cells do not express caspase-3, as described previously [29], [30]; thus, we examined caspase-7 and caspase-9 as the indicators of apoptosis. As shown in **Figure 3C**, concentration-dependent proteolytic fragments of caspase-7 and caspase-9 were observed after 48 h treatment of EVO.

### EVO Induced Apoptosis by Inhibiting Both the Ras/MEK/ERK Cascade and IAPs

**Figure 4:**
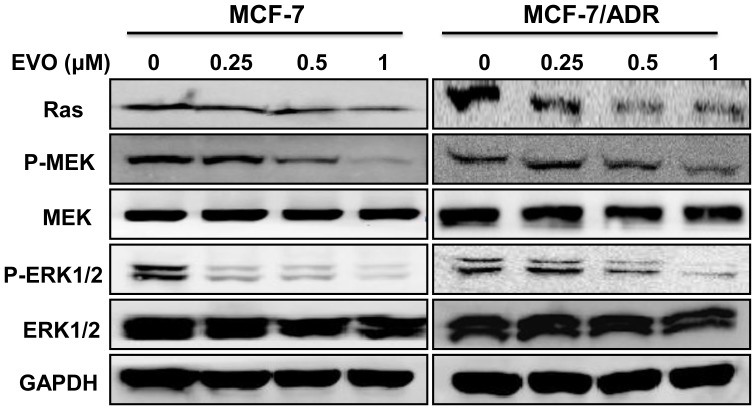
Inhibition of Ras/MEK/ERK signaling pathway in MCF-7 and MCF-7/ADR cells by EVO. Whole-cell lysates were generated and immunoblotted with antibodies against Ras, phosphorylated MEK (P-MEK), MEK, phosphorylated ERK (P-ERK1/2), ERK1/2 and GAPDH. Similar results were obtained in two or three separate experiments.

**Figure 5:**
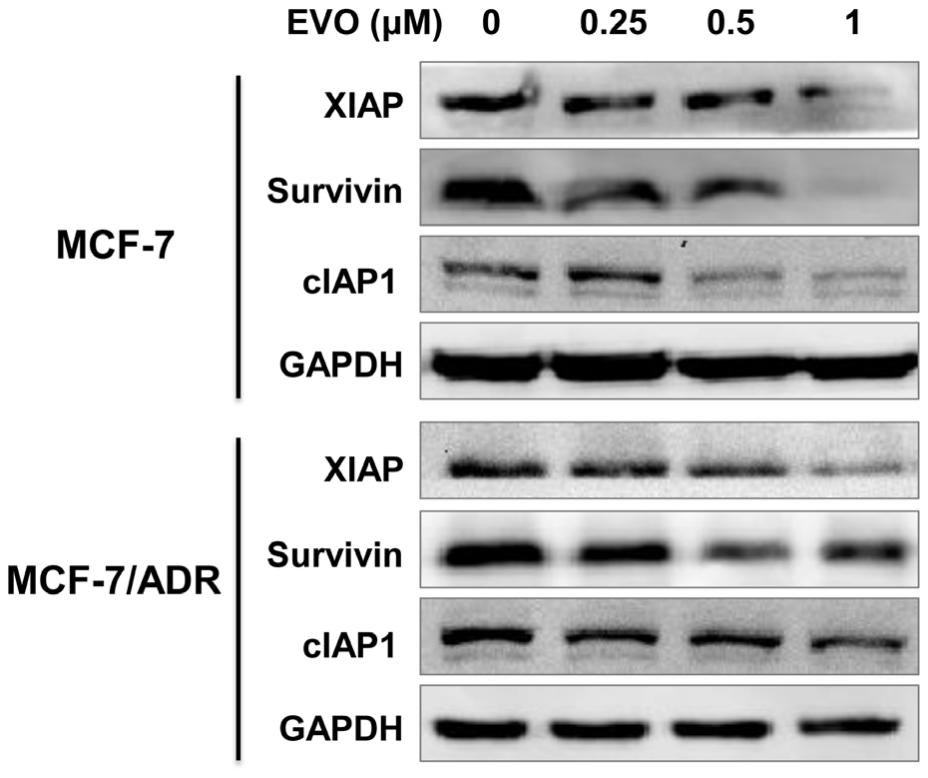
Inhibition of Inhibitor of Apoptosis (IAPs) in MCF-7 and MCF-7/ADR cells by EVO. Whole-cell lysates were generated and immunoblotted with antibodies against XIAP, Survivin, cIAP1 and GAPDH. Similar results were obtained in two or three separate experiments.

To elucidate the potential mechanisms underlying apoptosis induction by EVO, several proteins related to apoptosis were measured by Western blotting. Treatment of the cells with 0.25 to 1 µM EVO markedly inhibited the levels of Ras, P-MEK and P-ERK1/2, whereas the levels of total MEK and ERK1/2 protein were not affected significantly in either of the tested cell lines (**Figure 4**). We further examined the effects of EVO on the expression levels of IAP family proteins, including survivin, XIAP and cIAP1. Treatment of MCF-7 cells with EVO at 1 µM completely inhibited survivin, XIAP and cIAP1 expression. Moreover, marked inhibition of the levels of survivin, XIAP, and cIAP1 was also detected in the resistant breast cancer MCF-7/ADR cells (**Figure 5**).

### EVO Enhanced DOX Induced Growth Inhibition

**Figure 6:**
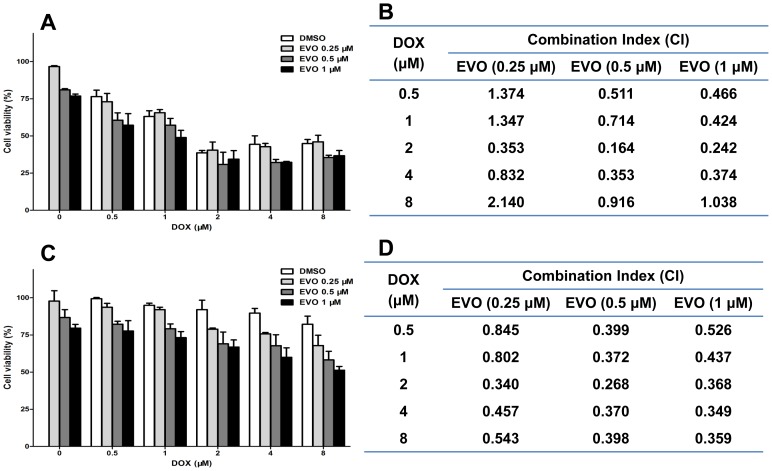
Combinational treatment of MCF-7 and MCF-7/ADR cells with EVO and DOX showed synergistic effect in reducing cell viability. MCF-7 and MCF-7/ADR cells were pretreated with different concentrations of EVO for 12 h, followed by treating with DOX for another 48 h. Cell viabilities of MCF-7 (**A**) and MCF-7/ADR (**C**) cells were measured by MTT assay. The combination index (CI) of EVO and DOX in MCF-7 (**B**) and MCF-7/ADR (**D**) cells were conducted using CalcuSyn software (Biosoft, Cambridge, UK), where CI<1 indicated synergistic effect. Data presented mean ± SE from three independent experiments conducted in triplicate.

The sensitizing cytotoxic effect of EVO in MCF-7 and MCF-7/ADR cells exposed to DOX is presented in **Figure 6**. At a concentration of 2 µM, 48 h DOX treatment inhibited MCF-7 cell viability by 60% but inhibited MCF-7/ADR cell viability by only 10%, indicating the resistance of MCF-7/ADR cells to DOX. However, when DOX was administered in combination with EVO, a significant decrease in cell viability was observed in both MCF-7 and MCF-7/ADR cells (**Figure 6A and 6C**). To characterize the interaction of these two agents, we analyzed the above results using the CalcuSyn program (Biosoft), which uses the Chou-Talalay Method, a derivation of the mass-action law principle [31]. The combination index (CI) is a quantitative measurement of the relationship between two agents, where a CI greater than 1 indicates antagonism and a CI less than one indicates synergism [32]. The CI for the interaction between DOX and EVO in MCF-7 and MCF-7/ADR cells were calculated and listed in **Figure 6B and 6D**. We observed that the sensitization effect of EVO in MCF-7/ADR cells was more significant than the sensitization effect in MCF-7 cells, as most of the CI values in MCF-7/ADR cells were less than those in MCF-7 cells.

### EVO Enhanced DOX-Induced Apoptosis in MCF-7/ADR Cells by Inhibiting IAPs and the Ras/MEK/ERK Cascade

**Figure 7:**
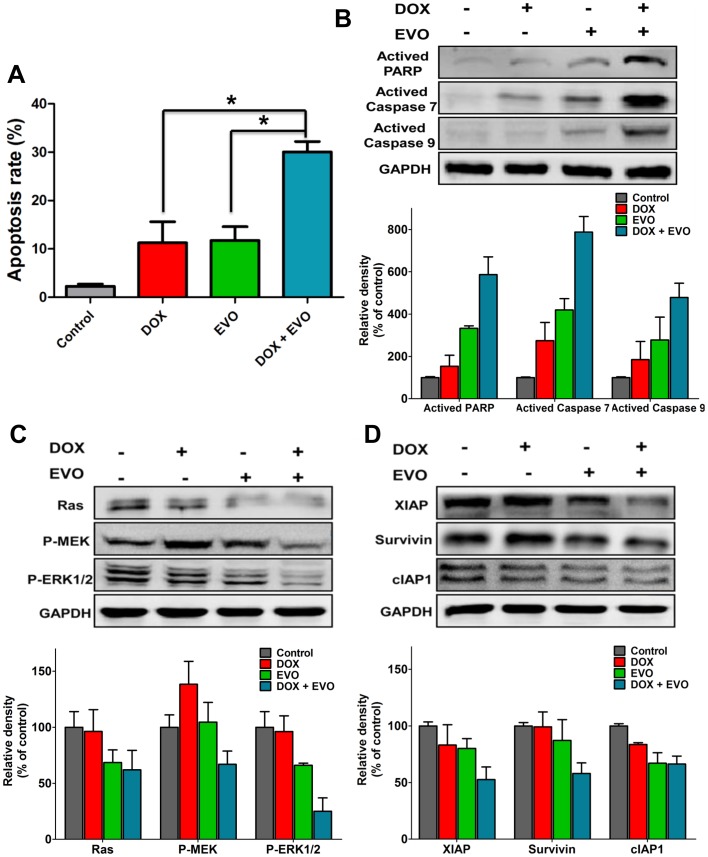
EVO enhanced DOX-induced apoptosis in MCF-7/ADR cancer cells. MCF-7/ADR cells were pretreated with EVO (1 µM) for 12 h, and then incubated with 2 µM of Dox for another 48 h. Apoptotic measurement was Annexin V/PI assay (**A**). Data are expressed as mean ± SE of three independent experiments. * P<0.05. (**B**) The cell lysates were generated for Western blot analysis using antibodies against activated PARP, activated caspase-7, -9 and GAPDH. Examination of the combined effects of DOX and EVO on expression levels of Ras/MEK/ERK cascade (**C**) and IAPs family proteins (**D**). Similar results were obtained in three separate experiments.

The combination of DOX and EVO resulted in a significantly higher percentage of apoptosis in MCF-7/ADR cells than either drug alone (**Figure 7A**). Individual treatment with DOX (2 µM) or EVO (1 µM) induced apoptosis in 11.27% and 11.77% of the cells, respectively. In contrast, concurrent treatment with DOX and EVO increased the apoptotic cell population to 30.05%. We then examined the status of PARP and caspases. As shown in **Figure 7B**, activated PARP and caspases were slightly or moderately induced by DOX or EVO as single agents, whereas the combination of DOX and EVO caused an obvious increase in the cleaved PARP, caspase-7 and caspase-9 levels, indicating that EVO sensitized MCF-7/ADR cells to DOX by inducing cell apoptosis. To further examine the mechanisms behind the combinatorial synergism, the roles of the Ras/MEK/ERK cascade and IAPs were examined in response to concurrent treatment. In MCF-7/ADR cells, combination treatment with DOX and EVO resulted in the marked decrease of Ras expression and the phosphorylation of MEK and ERK1/2 (**Figure 7C**). Furthermore, DOX and EVO potentiated the inhibitory effect on the expression of IAPs, including XIAP, survivin and cIAP1 (**Figure 7D**).

### EVO did not Increase the Intracellular Level of DOX and Exhibited Minimal Effect on P-gp Expression and Activity

**Figure 8:**
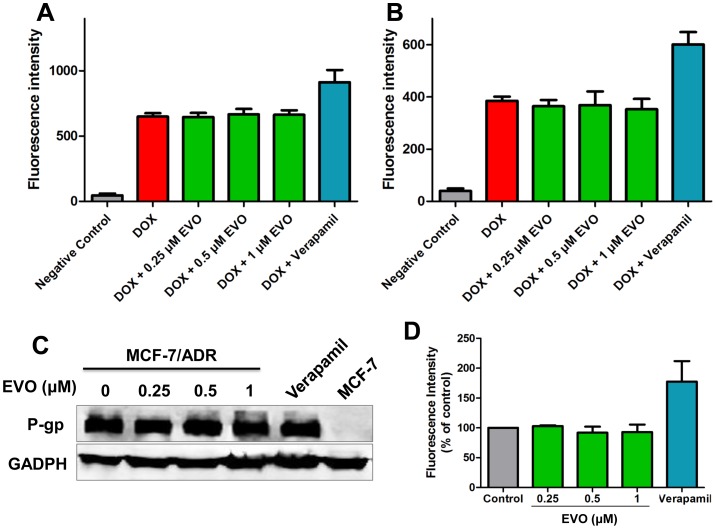
EVO sensitize the effect of DOX without inhibiting P-glycoprotein. MCF-7 (**A**) and MCF-7/ADR (**B**) cells were pretreated with EVO and Verapamil for 12 h, and then incubated Dox (2 µM) for another 4 h, then the intracellular level of Dox was determined using flow cytometry. (**C**) Effects of EVO on the expression levels of P-gp protein in MCF-7/ADR cells. After 24 h treatment of EVO and verapamil, protein levels in cell lysates were analyzed by Western blot. GAPDH was used as an internal control. Similar results were obtained in two or three separate experiments. (**D**) After 12 h treatment, the MDR pump activities were determined using a fluorimetric MDR assay kit (Abcam). Results are expressed as mean ± SE.

Flow cytometric analysis indicated that EVO did not augment the intracellular concentration of DOX in both MCF-7 and MCF-7/ADR cells (**Figure 8A and 8B**), unlike verapamil, a specific inhibitors of P-gp. We further investigated whether EVO could down-regulate the expression of P-gp and affect P-gp activity. As shown in **Figure 8C**, we observed that, over the 48 h treatment of EVO, the P-gp expression levels were not decreased. Furthermore, P-gp activity was examined using a fluorometric MDR assay kit in DOX-resistant cells (**Figure 8D**). No difference in the mean intracellular fluorescence value was observed between cells pretreated with EVO for 12 h compared with untreated cells, where greater numbers suggest more inhibitory effects on the P-gp activity.

## Discussion

To date, in many cases, there remains no remedy to overcome drug resistance and improve clinical outcomes in resistant cancers [33]. Many attempts have been made to inhibit membrane transporters, however, most of these drug transporter inhibitors have not proven to be effective in the clinic [11]. Therefore, more specific, targeted and low-toxicity therapies have attracted the interest of researchers as promising approaches to kill resistant cancer cells. The IAPs play key roles at the intersection of the mitochondria pathway and death receptor pathway and widely and potently inhibit apoptosis against multiple apoptotic stimuli, including chemotherapeutic agents and radiation [34]. Therefore, IAPs have been proven to be closely related to therapy resistance, and strategies targeting IAPs may be effective for overcoming apoptosis resistance [35]. For example, overexpression of XIAP can increase resistance to tumor necrosis factor-related apoptosis-inducing ligand (TRAIL), whereas down-regulation of XIAP restored the cell response to TRAIL [36]. Meanwhile, chemotherapeutic drugs such as DOX and docetaxel can induce the activation of the Ras/MEK/ERK pathway, and the activated cascade may regulate downstream factors that are involved in DNA repair and apoptosis, thereby contributing to drug resistance [11]. Therefore, synchronously targeting IAPs and the Ras/MEK/ERK pathway may prevent drug resistance and the reemergence of cancer initiating cells.

Many plant-derived compounds are known to enhance the chemotherapeutic effects of anticancer agents by modulating the main ABC (ATP-binding cassette) transporters responsible for cancer drug resistance, including P-gp, MRPs and BCRP, as well as regulating both cell survival- and cell death-related signaling pathways, which makes them a promising group of low toxicity candidates for reversing MDR [37]. As EVO has been reported to inhibit the expression of IAPs, such as XIAP, survivin and cIAP1 [19], and to modulate ERK signaling [21]–[23], we hypothesized that EVO could reverse DOX-resistant breast cancer cells by inhibiting the Ras/MEK/ERK pathway and IAPs.

In the present study, we observed that EVO significantly inhibited the proliferation and promoted the cytotoxicity on both DOX-sensitive MCF-7 cells and DOX-resistant MCF-7/ADR cells in a time- and concentration-dependent manner, as confirmed by MTT and LDH assay. We also observed that EVO induced apoptosis in MCF-7 and MCF-7/ADR cells in a caspase-dependent manner, as confirmed by the elevated levels of PARP, caspase 7 and caspase 9 as well as a significant increase in caspase activities. Furthermore, treatment of the cells with 0.25 to 1 µM EVO markedly inhibited the levels of Ras, P-MEK and P-ERK1/2 and decreased the expression levels of IAP family proteins, including survivin, XIAP and cIAP1, in both MCF-7 and MCF-7/ADR cells.

Cancer cells can abnormally activate survival pathways and upregulate the expression of IAPs after chemotherapy agent treatment, potentially preventing the downstream apoptotic responses and decreasing the sensitivity of the cells to these agents [38]. In the present study, MCF-7 cells were more sensitive to EVO than MCF-7/ADR cells, as EVO treatment resulted in greater growth inhibition, cytotoxicity and apoptosis and inhibited the Ras/MEK/ERK pathway and IAP expression. These effects may be attributed to the activation survival pathways and apoptosis resistance induced by DOX in chemotherapy. Meanwhile, because Ras/MEK/ERK pathway is normally activated and the levels of IAPs are quite low in normal cells, EVO may selectively induce cancer cell death, which further facilitates its application in the clinic.

The one-dimensional mechanism of action of single-drug chemotherapy often activates and heightens alternative pathways, thereby prompting the emergence of chemoresistance mutations and tumor relapse [39]–[42]. Synergistic combination of two or more drugs is an effective strategy for targeting both apoptosis resistance and increased survival signaling to overcome chemoresistance. DOX is commonly used as a first-line chemotherapeutic agent for the treatment of breast cancer. DOX can incorporate into the DNA of cancer cells and prevents cell replication by suppressing protein synthesis [43]. However, unwanted side effects and the development of drug resistance limit its application. Therefore, increasing the sensitivity to DOX is an attractive strategy for improving the clinical management of breast cancer [44]. In this study, we observed that DOX and EVO exhibited a synergistic effect in the induction of apoptosis in MCF-7/ADR cells, which was evidenced by the presence of cleaved caspase-7, caspase-9 and PARP. The ability of EVO to block both the Ras/MEK/ERK pathway and IAP expression may sensitize human cancer cells to chemotherapy. In our experiment, we demonstrated that treatment with DOX had little effect on the Ras/MEK/ERK pathway and IAP expression, whereas the combination of DOX and EVO led to the decreased expression of Ras, P-MEK, P-ERK1/2, XIAP, survivin and cIAP1 simultaneously. We believe that EVO inhibition of the Ras/MEK/ERK pathway and IAP expression may have led to the higher levels of caspase activity observed in the resistant cancer cells after the combination treatment, thus significantly enhancing the effects of DOX.

The development of MDR is frequently associated with the overexpression of ATP-dependent membrane transporters like P-gp and MRP1, which actively pump out cytotoxic drugs from cancer cells, thus reducing their intracellular concentration and efficiency[45]. Previous studies have reported that EVO could inhibit TNF-induced expression of P-gp in KBM-5 cells [19]. However, in our present study, though obviously higher cytotoxicity was observed when DOX was combined with EVO, further studies demonstrated that EVO did not result in the down-regulation of P-gp expression or the severe impairment of its function. The effects of EVO on P-gp expression may be cell-type specific and may be related to the expression levels in targeted cancer cells. These results indicate that the sensitization mechanism of EVO to DOX was independent of P-gp inhibition, at least under the experimental conditions presently employed.

Taken together, our data indicate that EVO is proapoptotic, reverses drug resistance and acts synergistically with DOX by inhibiting both IAPs and the Ras/MEK/ERK cascade without inhibiting P-gp. As a natural product, EVO, used alone or in combination with chemotherapeutic agents, may be useful in the treatment of resistant breast cancer.
